# Combined stress of ocean acidification and warming influence survival and drives differential gene expression patterns in the Antarctic pteropod, *Limacina helicina antarctica*

**DOI:** 10.1093/conphys/coaa013

**Published:** 2020-03-26

**Authors:** Kevin M Johnson, Gretchen E Hofmann

**Affiliations:** 1 Department of Biological Sciences, Louisiana State University, Baton Rouge, LA 70803 USA; 2 Department of Ecology, Evolution and Marine Biology, University of California, Santa Barbara, Santa Barbara, CA 93106-9620 USA

**Keywords:** *Limacina helicina antarctica*, ocean acidification, ocean warming, physiological plasticity, Pteropod, RNAseq

## Abstract

The ecologically important thecosome pteropods in the *Limacina spp.* complex have recently been the focus of studies examining the impacts global change factors – e.g., ocean acidification (OA) and ocean warming (OW) – on their performance and physiology. This focus is driven by conservation concerns where the health of pteropod populations is threatened by the high susceptibility of their shells to dissolution in low aragonite saturation states associated with OA and how coupling of these stressors may push pteropods past the limits of physiological plasticity. In this manipulation experiment, we describe changes in the transcriptome of the Antarctic pteropod, *Limacina helicina antarctica*, to these combined stressors. The conditions used in the laboratory treatments met or exceeded those projected for the Southern Ocean by the year 2100. We made two general observations regarding the outcome of the data: (1) Temperature was more influential than pH in terms of changing patterns of gene expression, and (2) these Antarctic pteropods appeared to have a significant degree of transcriptomic plasticity to respond to acute abiotic stress in the laboratory. In general, differential gene expression was observed amongst the treatments; here, for example, transcripts associated with maintaining protein structure and cell proliferation were up-regulated. To disentangle the effects of OA and OW, we used a weighted gene co-expression network analysis to explore patterns of change in the transcriptome. This approach identified gene networks associated with OW that were enriched for transcripts proposed to be involved in increasing membrane fluidity at warmer temperatures. Together these data provide evidence that *L.h.antarctica* has a limited capacity to acclimate to the combined conditions of OA and OW used in this study. This reduced scope of acclimation argues for continued study of how adaptation to polar aquatic environments may limit the plasticity of present-day populations in responding to future environmental change.

## Introduction

In a global change context, experimental biologists have been tackling the challenging issue of assessing responses of marine organisms to multiple stressors (Breitburg, 2015, Gunderson *et al.*, 2016, Harvey *et al.*, 2013, Stillman *et al.*, 2013). This area of research is complex due to the many interactions of the stressors with biological traits, along with other contexts such as the evolutionary, and/or environmental history of the organism under-study. With regard to marine invertebrates, there is a growing body of evidence that the biological response to multiple stressors is variable within taxa and between life-history stages (Byrne, 2011, Haigh *et al.*, 2015, Harvey, Gwynn-Jones and Moore, 2013, Przeslawski and Byrne 2013). In addition, the observed responses in any one taxa might fall into various categories; specifically, additive effects (the influence of each combined stressors is equal to the degree of stress produced by that stressor alone), synergistic effects (wherein the influence of the combined stressors is greater than the sum of the stressors), or antagonistic effects (wherein the influence of the combined stressors is less than the sum of the stressors). Overall, conservation physiologists have realized that understanding the complex responses to multiple stressors will be key to our ability to accurately predict the resilience, tolerance, and adaptive potential of a species in response to environmental changes that are typically associated with climate change (Calosi *et al.*, 2016, Kelly *et al.*, 2016, Reusch, 2014, Stillman and Armstrong, 2015, Stillman and Todgham, 2013). In this study, we examined the transcriptomic response of an ecologically important member of the Southern Ocean macrozooplankton community in response to two abiotic stressors associated with anthropogenic climate change: ocean acidification (OA) and ocean warming (OW) (Calosi *et al.*, 2016, Harvey *et al.*, 2013, Reusch, 2014).

Studying the effects of OA and OW on marine invertebrates is important as both of these stressors are associated with increasing levels of atmospheric CO_2_: OA results when CO_2_ dissolves into surface oceans, reducing ocean pH, while OW is driven by the increasing air temperatures due to the greenhouse effect of CO_2_ (Boyd *et al.*, 2014, Schnoor, 2014). While the effects of these combined stressors has been shown to be highly variable in molluscs (Stevens and Gobler, 2018), controlled laboratory experiments are providing valuable insights as to the overall susceptibility of indicator species across a range of marine ecosystems (Constable *et al.*, 2015, Deutsch *et al.*, 2015, Haigh *et al., 2015*). These multi-stressor approaches have highlighted significant variation in response patterns at the level of taxa. For example, tropical stony corals are thought to be highly susceptible (Pandolfi *et al.*, 2011) while organisms from more variable, temperate marine environments such as tidal flats or upwelling regions generally display a greater range of tolerances (Evans *et al.*, 2017, Stevens and Gobler, 2018, Wong *et al.*, 2018). Some groups of organisms, such as calcifying marine invertebrates from high latitude seas, are thought to be more vulnerable due to the already low pH nature of cold polar seas (Gardner *et al.*, 2017, Johnson and Hofmann, 2017, Lischka *et al.*, 2011, Manno *et al.*, 2017), and the tendency of these animals to have an evolutionary history that appears to limit physiological plasticity in response to environmental variability (Peck *et al.*, 2010, Peck *et al.*, 2014, Peck *et al.*, 2004).

With regard to OA, among the most vulnerable of species identified to date are the thecosome pteropods in the *Limacina helicina spp.* complex that inhabit both the Southern and Arctic Oceans (Bednaršek *et al.*, 2017). These small pelagic molluscs have been collected *in situ* with signs of significant shell damage (Bednaršek *et al.*, 2017, Bednaršek *et al.*, 2012, Johnson *et al.*, 2016), likely in response to present day low pH/under-saturated conditions. In addition, extensive shell dissolution and mortality has been observed in laboratory experiments that mimic OA and OW conditions (Lischka *et al.*, 2011). This response to combined stressors suggests that the ability for pteropods to persist may be dependent on their present-day degree of phenotypic and physiological plasticity. Therefore, establishing the degree of plasticity in response to these combined stressors can provide important insights into the potential for genetic capacity that could allow a species to persist, and potentially adapt to the changing abiotic conditions (Kelly, 2019).

In this study, we focused on the transcriptome as a molecular-level physiological response of the thecosome Antarctic pteropod *Limacina helicina antarctica* to the combination of pH and temperature stress. The use of acute comparative studies in manipulative microcosom experiments is widely used to compare the physiological response of organism’s to environmental drivers (e.g. pH and temperature; Riebesell and Gattuso, 2015; Boyd *et al*., 2018). Previous research using similar experimental designs that mimicked OA and OW on *L. h. antarctica* larval survival, calcification, and shell morphology has highlighted that the combined stress of warming (+3.5 °C) and acidification (pH 7.60) led to increased mortality, increased shell dissolution, and decreased growth rates in a non-additive manner (Gardner *et al.*, 2017). In addition, in a study conducted in parallel to the study presented here, Hoshijima *et al.* (2017) identified additive effects of warming (+4 °C) and acidification (pH 7.69) leading to increased respiration rates in young adult *L. h. antarctica* individuals. Interestingly, there was almost no change in respiration rate between pH treatments following 24 hours of exposure for each temperature, suggesting temperature stress may dominate the acute physiological response, and the influence of OA only appears during the acclimation response window. In contrast, examining a different set of traits, Gardner *et al.* (2017) did not detect additive effects of these combined stressors on larval mortality or shell morphology. These results suggest that aragonite undersaturation regardless of temperature is the primary driver of mortality and shell dissolution, while the influence of the combined stressors is only apparent when assessing physiological responses in young adults. Similar age-dependent thermal tolerance in Antarctic marine invertebrates has previously been observed and suggests that early life-history stages may be more resilient to ocean warming (Peck *et al.*, 2013). In addition, while pteropods are believed to live up to 3 years (Wang *et al.,* 2017); the occurrence of adult pteropods (e.g., larger then 1 mm and with mature gonads) have not been found during the early spring in the McMurdo Sound (Johnson *et al.,* 2016). Previous research into transcriptomic changes across season has highlighted large transcriptome-wide changes in gene expression associated with developmental progression and food availability (Johnson *et al*., 2018). Together, these studies suggest that in acute responses, OW drives physiological responses, but that OW combined with OA exasperates the physiological demands of increased temperatures over time.

Previous comparative transcriptomic studies of juvenile *L. h. antarctica* have focused on the single driver of OA via the manipulation of *p*CO_2_ in experimental mesocosms and with field collections that explored changes in gene expression between seasons (Johnson and Hofmann, 2016, Johnson and Hofmann, 2017, Johnson *et al.*, 2018, Koh *et al.,* 2015). Here we report the molecular response to the combination of pH and temperature stress utilizing comparative transcriptomics in a multi-stressor experiment wherein we exposed pteropods to 3 pH conditions (pH 8.10, 8.01, and 7.71) at an elevated temperature (+4 °C). We also explore how these results compare to our previously published study (Johnson and Hofmann, 2017) that compared 3 similar pH conditions (pH 8.07, 7.95, and 7.70) at an ambient temperature (−1.0 °C) and measured changes in gene expression over time (12 hours up to 10 days). The pH treatments used for both scenarios are based on projections for acidification in the Southern Ocean (Kapsenberg *et al*., 2015); while the elevated temperature of +4 °C was chosen to provide a significant thermal challenge and be comparative to other multistressor experiments (IPCC, 2013; Hoshijima *et al.,* 2017; Gardner *et al.,* 2017). We also examined the acute molecular response to these combined stressors using both an analysis of deviance and a weighted gene network analysis to examine gene expression patterns associated with pH, temperature, and aragonite saturation state (Ω_aragonite_). These data provide additional evidence that *L. h. antarctica* will be significantly impacted by the combined stress of OA and OW in the Southern Ocean.

## Materials and methods

(a) *Pteropod collections*

Juvenile *Limacina helicina antarctica* were collected in McMurdo Sound in the southern Ross Sea during a 2015–2016 summer field season (supported by the U.S. Antarctic Program and a grant from the U.S. National Science Foundation). Using plankton net tows through a hole in the sea ice to gain access to the water column, pteropods were collected at a depth of 50 meters using a fixed-frame bongo net (50 cm x 150 cm net with 333 μm mesh and cod ends with 200 μm and 333 μm mesh). The bongo net was deployed through a dive hole in the sea ice at a near-shore site on the sea ice (Fish Hut 03; 77°50′53” S, 166°35′59″ E), and pteropods were collected for two experiments: one conducted in October 2015 at ambient temperatures (−1.2 °C) hereafter referred to as “A” and thoroughly described in Johnson and Hofmann (2017); and, another experiment conducted three weeks later in November 2015 in at an elevated temperature of +4 °C, hereafter referred to as “E”. After collection, pteropods were maintained in ambient seawater (−1.7 °C) in small Nalgene containers, and were transported to the Crary laboratory facilities at McMurdo Station, the U.S. research station on Ross Island, Antarctica. Upon arrival, 9 groups of 800 actively swimming individuals were hand sorted in a temperature-controlled room (−2 °C) and maintained at −1.7 °C for 24 hours prior to use in the A and E manipulation experiments. At the start of each experiment 1 cohort of 800 swimming individuals was haphazardly chosen and added to each of 9 pre-equilibrated treatment tanks (3 tanks per treatment).

(b) *Treatment conditions*

**Figure 1:**
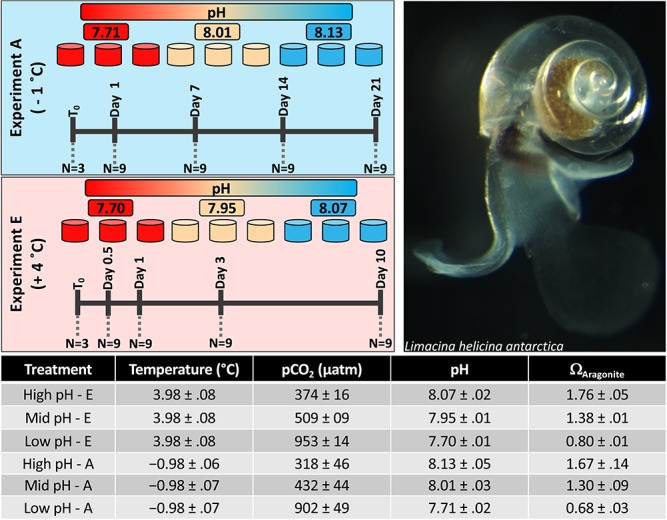
Treatment conditions and sampling time-points for each of the Ambient and Elevated temperature experiments. Values reported represent means ± standard deviation. Ambient temperature conditions were also previously reported (see: Johnson and Hofmann, 2017).

In each of the two temperature experiments (A and E) there were 3 OA treatment conditions representing a high, mid and low pH level. In each run there was some variability in the precise pH maintained during the experiment. The pHs for experiments A and E are as follows: high pH: pH 8.07 for A and pH 8.10 for E; mid pH: pH 7.95 for A and pH 8.01 for E; low pH pH 7.70 for A and pH 7.71 for E (Figure 1). These pH levels were maintained using a CO_2_ mixing system that controlled *p*CO_2_ in reservoir tanks that then fed replicate tanks holding the pteropods; this system has been previously described (Johnson and Hofmann 2017). For each OA condition there were 3-replicate 1.5 L tanks equipped with 2 L/h irrigation drippers. This provided a high turnover rate within each tank allowing for low variability in the experimental conditions throughout the 10-day elevated temperature experiment (Figure 1). For experiment A, we used 5 sampling time-points: Time 0 (immediately prior to the addition of individuals to the experimental tanks), and at Days: 1, 7, 14 and 21. For experiment E, we used 5 sampling time-points: Time 0, 12 hours after addition to the tanks, and then at Days: 1, 3, and 10. In order to compare the effect of temperature we used individuals collected at Day 1 from the previously published OA only experiment (experiment A; Johnson and Hofmann, 2017). Due to differences in sampling time points between experiment’s E and A we have restricted the comparison between experiments to only the samples that reflect the same duration of exposure in both experiments (i.e. Time 0 and Day 1, see Figure 1). For each tank, at each sampling time-point, 3 tubes of 10 actively swimming pteropods were placed into a 1.5 ml micro-centrifuge cryovial, excess water removed, and the sample flash frozen in liquid nitrogen before being stored at −80 °C. In addition to these 5-sampling time-points, mortality checks were conducted daily on all tanks, and all deceased individuals were removed. Mortality was assessed on non-swimming individuals using visual observation of activity through their translucent shells. The effect of pH treatment on mortality for experiment E was tested with a Cox proportional hazards regression model (Hoshijima *et al.,* 2017).

Seawater chemistry was performed as previously described (Johnson and Hofmann 2017). In brief, seawater chemistry was measure for each of the 9 experimental tanks. Total alkalinity was measured every 3 days with water samples collected from each header tank, poisoned with 0.02% mercuric chloride, and stored at +4 °C until analyzed (Dickson *et al.*, 2007). Total alkalinity measurements were conducted using an open-cell titration method and pH readings were conducted using the m-cresol spectrophotometric method Clayton and Byrne (1993) . In addition, daily temperature, pH, and salinity measurements were collected for each header tank and each of the 9 experimental holding tanks. From these measurements carbonate chemistry parameters were calculated using CO2Calc (Robbins *et al.*, 2010).

(c) *RNA extraction and library preparation*

For both experiments, only individuals that were actively swimming in the water column were collected for differential gene expression analysis. RNA was extracted from 3 pools of 10 pteropods collected from each replicate OA treatment tank (n = 3 per treatment). Extractions were performed using 500 ul of Trizol® reagent according to the manufacturer’s instructions (Invitrogen). For each sample, total RNA was analyzed for quantity and purity using the NanoDrop® ND100; and RNA integrity assessed by electrophoresis using the Tapestation 2200 system (Agilent Technologies). All samples used for library preparation had a RINe scores above 8.5. In total, 39 separate RNAseq libraries from each temperature treatment were generated using the Illumina TruSeq Stranded mRNA Library Preparation Kit. During the preparation, the libraries were amplified using 12 cycles of PCR. Completed libraries were screened for mean lengths of 428 bp (300 bp insert +128 bp sequencing adapters) using the Agilent Tapestation. Concentrations of each final library were quantified using a Qubit® 3.0 flourometer (Life Technologies). All libraries were sequenced in 4 pools on four lanes of a HiSeq4000 Illumina sequencer using paired-end 100 base-pair reads, 2 lanes for experiment E and 2 lanes for experiment A. Sample pooling and sequencing were performed at the UC Davis Genome Center (https://genomecenter.ucdavis.edu).

Paired sequencing reads were trimmed of adapters and low-quality base pairs (Phred <20) using the program Trimmomatic (v.0.36) (Bolger *et al.*, 2014). Trimmed reads were then mapped to the previously published transcriptome using RSEM (Johnson and Hofmann, 2016, Li and Dewey 2011). This transcriptome was developed from juvenile pteropods exposed to either i) no treatment, ii) 1 of 3 pH treatments; or iii) an acute elevated temperature stress (+8 °C; Johnson and Hofmann (2016)). The high temperature experiment (E), mapped reads were imported into R and were filtered using the R program edgeR so that only sequences with more than 0.5 counts per million (cpm) in 9 of the 39 libraries were retained and the count data normalized using the edgeR_robust pipeline (Zhou *et al.*, 2014). Global changes in gene expression were assessed using edgeR with the filtered read counts log-transformed using the *cpm* function and setting prior.count equal to 2. These log-transformed counts were used to conduct a principle coordinate analysis (PCoA) using manhattan distances calculated with the *pcoa* function as implemented in the R program vegan (Oksanen *et al.*, 2018).

In order to explore gene expression changes between the E experiment and the previously published A experiment (Johnson and Hofmann, 2017), all reads from the 24-hour sampling time point from both experiments (9 samples per temperature) were imported into R and filtered so that only sequences with more than 0.5 cpm in 12 of the 18 samples were retained. With these data, we used a permutational multivariate analysis of variance to assess the effects of temperature + pH on gene expression using 10^6^ permutations within the *adonis* function in the R program vegan (Oksanen *et al.*, 2018).

(d) *Differential gene expression analysis*

Differential gene expression (DGE) analysis for the multi-stressor experiment was conducted with edgeR in three ways: (1) Using pairwise comparisons between each time-point and Time 0 for all samples in experiment E regardless of pH treatment, (2) between all experiment E pH treatments at each time-point (see supplemental file 1), and (3) between the A and E experiments comparing samples collected after 24 hours of incubation. Additional comparisons between all of experiment A’s pH treatments have previously been reported in Johnson and Hofmann (2017). This approach allowed us to identify genes that are (1) significantly changing in response to high temperature as a function of time, regardless of pH treatment, (2) genes that are changing in expression response to pH treatment at each time-point, and (3) genes that are changing in response to pH and temperature at day 1. For comparisons 1 and 2, a single model was generated using all experiment E samples without providing an intercept allowing for all pairwise comparisons to be made from a single contrast matrix. For comparison 3, a model combining the 9 samples from experiments E and A (restricted to Time 0 and Day 1 from both experiments) was generated without an intercept to allow all comparisons to be made with this single contrast matrix. Differentially expressed genes identified in each of these analyses were assessed for gene ontology and KEGG enzyme term enrichment using a Fisher’s exact test (FDR < 0.05) as implemented in the software Blast2GO.

**Table 1:** Differential gene expression between each sampling timepoint and the pre-exposure Time 0 sample.

Sequence ID	12 Hour (LogFC)	Day 1 (LogFC)	Day 3 (LogFC)	Day 10 (LogFC)	FDR	Description	Gene Ontology Term
TR609897|c1_g1_i4	12.31	9.97	8.25	11.34	0.013	PREDICTED: uncharacterized protein LOC101854471	Lipid transport
TR391802|c1_g1_i2	1.53	2.18	3.04	2.26	0.0023	Mitochondrial import inner membrane translocase subunit TIM44-like	Chaperone binding
TR559229|c4_g1_i25	4.20	2.45	8.40	5.21	0.0001	Heat shock 70 kDa cognate 5	Unfolded protein binding
TR522115|c1_g2_i2	1.49	1.88	2.36	1.88	0.0002	Parkin coregulated gene homolog	HSP90 protein binding
TR581794|c2_g1_i8	4.17	2.87	6.92	5.94	0.04991	Chorion peroxidase-like	Oxidoreductase activity
TR601318|c3_g1_i10	1.69	3.04	4.08	5.05	0.00217	Mitochondrial ribosomal L23	Structural constituent of ribosome
TR542716|c1_g4_i2	1.30	1.86	1.58	2.13	1.17E-05	Allene oxide synthase-lipoxygenase -like	Oxidation-reduction process
TR560734|c2_g1_i2	4.07	8.70	10.82	10.77	1.13E-5	Actin 6B	RNA polymerase II proximal promoter sequence-specific DNA binding
TR532393|c2_g1_i1	2.75	2.77	2.58	2.44	0.01119	Peptidyl-prolyl cis-trans isomerase H	Peptidyl-prolyl cis-trans isomerase activity and protein folding
TR590246|c1_g1_i8	−3.96	−6.84	−2.91	−9.07	0.02059	N-terminal asparagine amidohydrolase	Hydrolase activity
TR550939|c2_g1_i4	−4.37	−3.16	−11.71	−11.71	0.00208	Testis-specific serine threonine- kinase 2	Protein serine/threonine kinase activity
TR587359|c0_g1_i1	−9.31	−13.82	−11.52	−12.45	0.00000	Cytochrome c oxidase subunit I	Ion binding
TR429485|c0_g2_i4	−1.63	−1.06	−2.66	−4.77	0.00007	Sodium potassium calcium exchanger 2- partial	Ion binding
TR535972|c1_g2_i3	−0.31	−0.73	−2.52	−2.73	0.00000	Importin 9	Ran GTPase binding
TR533884|c3_g1_i2	−1.29	−2.56	−3.83	−4.91	0.00002	Tribbles homolog 2-like	Transcription corepressor

To further explore the data set, two additional DGE analyses were conducted with the A and E experiments following 24 hours of incubation; these approaches were: (1) An ANODEV in edgeR to identify gene expression patterns associated with temperature for each pH treatment, but not between pH treatments at differing temperatures, and (2) a weighted gene co-expression network analysis (WGCNA) Langfelder and Horvath (2008) to identify gene co-expression networks associated with acute temperature by pH exposure. For the WGCNA analysis the count matrix was further filtered using edgeR to remove lowly expressed transcripts with less than 2 cpm in 9 of the 18 samples. Following this filtering, the WGCNA analysis was completed using a signed hybrid network analysis with a soft threshold power of 8 and a minimum module size of 30 genes. Each module was further clustered using a cut height of 0.30 as determined using the *dynamicMergeCut* function in WGCNA. The resulting modules were correlated with each of the treatment conditions and GO enrichment tested using the fishers exact test as implemented in Blast2GO. All codes for differential expression analysis are available in supplemental file 4.

**Figure 2:**
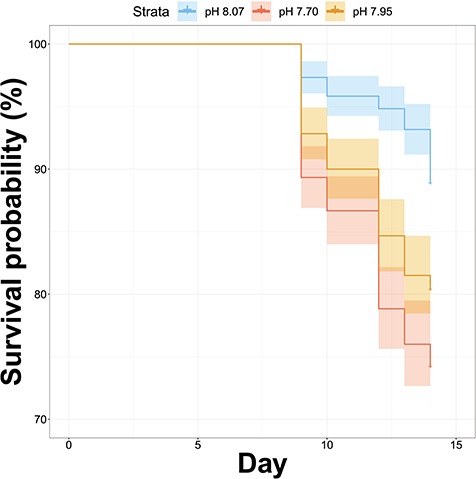
Survival Plot highlighting effect of pH treatment during the course of experiment E (+4 °C). Shaded regions represent 95% confidence intervals around the mean.

## Results and Discussion

(a) *Survivorship during the 10-day experiment*

Conditions of the seawater throughout the experiment were highly stable with low variability in pH, *p*CO_2_, total alkalinity, temperature, and salinity (Table 1). In terms of mortality for experiment E, there was 100% survivorship across all treatments through the first 8 days of the sampling period. However, on day 9 there were significant mortalities in all three pH treatments, and these losses continued into day 14 (supplemental file 1). A Cox proportional hazards regression model of mortality by treatment for experiment E revealed a significant effect of pH treatment (p-value = 6.245e-11; Hoshijima *et al.* 2017). This model was only run using mortality data from experiment E because the ambient temperature experiment (experiment A) did not produce significant mortality for any pH treatment when pteropods were held for 21 days (Johnson and Hofmann, 2017).These observed patterns in mortality highlight that increased temperature is the primary driver of mortality, but that the combination of low pH conditions with elevated temperatures significantly impacts survivorship (Figure 2). These differences suggest pteropods have a reduced ability to meet the energetic demands generated under OA conditions when experienced in conjunction with elevated temperature.

**Figure 3:**
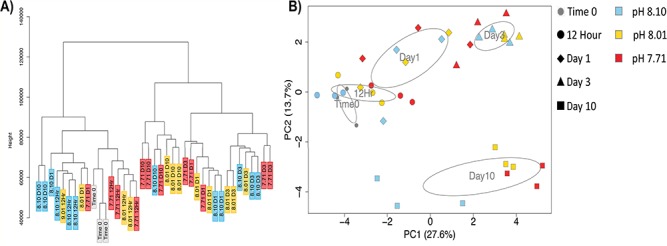
A) Hierarchical clustering of gene expression for individuals cultured at +4 °C. B) Biplot of the first two principle components, sample day is represented by icon shape while pH is designated by color.

The initiation of mortality across treatments at Day 9 further support the hypothesis that temperature is the major contributor to decreased survival as mortality for all treatments began to appear during the same day (Figure 2). However, one major consideration when viewing these mortality data is that these incubation experiments did not include active feeding, and it is very probable that if such an experiment were to be conducted again with active feeding these temperature-associated mortality events might be reduced. Previous research in the OA and OW fields have shown that increased feeding may offset the effects of these combined stressors (Chan *et al.*, 2011, Matzelle *et al.*, 2015, Saba *et al.*, 2012). In the experiments described here, feeding was not included due to uncertainties as to what an appropriate food source would be and noting that non-endemic species would not be approved for release in the Antarctic field location. In addition, a recent meta-analysis by Clements and Darrow (2018) proposed that OA and OW may in fact drive higher feeding rates in calcifying marine invertebrates.

(b) *RNA-sequencing*

RNA sequencing of the 39 elevated temperature libraries resulted in 737 million reads with approximately 18.9 million reads per sample (range: 10.2–25 million reads per sample). RSEM mapping of these trimmed reads resulted in 71.75% (range: 63.49–77.37%) of sequence reads mapping to the previously published transcriptome (Johnson and Hofmann 2016). Filtering transcripts to only those that exceeded 0.5 counts per million reads in at least 9 samples produced a final counts matrix consisting of 78 326 transcripts with an average expression of 11.1 million reads per sample (range: 6.4–17.4 million reads per sample). Sequencing of the previously reported 39 ambient temperature libraries produced 803 million reads (range: 15.4–24.7 million reads per library), RSEM mapping and trimming resulted in 71% of sequences being successfully mapped (range: 67.5% – 73.8%; Johnson and Hofmann, 2017).

(c) *General patterns: Principle coordinate analysis*

In general, there were significant changes in global gene expression among animals as a function of temperature and time in the experimental tanks. Specifically, for animals maintained at +4 °C in the E experiment, principle coordinate analysis (PCoA) identified significant changes associated with pH treatment (Adonis p-value = 0.00086) and sampling time point (Adonis p-value = 1e-06), and a nearly significant effect of pH:time (Adonis p-value = 0.051). Analysis of pair-wise DGE identified increasing levels of differential expression throughout the course of the experiment. In addition, there were strong per-day effects with less sample variation between treatments as compared to between days (Figure 3B –PCA). In total, 14 073 transcripts associated with 437 gene ontologies, that were differentially expressed throughout the experimental incubation (supplemental file 2). While the magnitude of differential gene expression increased over time, there was only minimal overlap between genes differentially expressed between treatments at each of the different time points. As such changes in expression over time may also reflect the effects of food limitation while acclimating to elevated temperature and pH; and therefore may reflect changes in broad metabolic processes.

Overall, these global patterns of gene expression indicate that Antarctic pteropods possess a significant degree of transcriptomic plasticity, and thus appear to have a compensatory mechanism to respond to acute changes in their abiotic, thermal environment. This capacity may be ecologically significant as pteropods are known vertical migrators, and as members of the macrozooplankton community face a dynamic abiotic environment *in situ* (Comeau *et al.*, 2012, Manno *et al.*, 2017). Such phenotypic plasticity to abiotic perturbations has been examined in other Antarctic ectotherms with varied results, although many studies demonstrate that Antarctic marine ectotherms do have the capacity to respond to abiotic changes, despite being characterized as stenothermic, or narrowly-adapted organisms (Barnes *et al.*, 2010, Peck *et al.*, 2013, Pörtner *et al.*, 2007). In our study, the degree of variation in gene expression provides evidence that pteropods were continually modifying their transcriptome in response to elevated temperature in all the pH treatments. These responses, when viewed in conjunction with the observed increased rates of mortality, suggest that even when pteropods were maintained under lab conditions that mimicked future OA, they were still able to modulate their gene expression patterns even in response to extreme conditions that are that are not likely to be encountered encountered in their present-day habitat in the Southern Ocean, but that are expected to occur regularly in the next 20–30 years during the winter months when seawater is at its lowest saturation state and highest *p*CO_2_ (Hauri *et al.*, 2016, Kapsenberg *et al.*, 2015). This plasticity is quite important in the near-term for Antarctic zooplankton. Specifically, a recent study notes that the depth of the saturation state horizon for aragonite is becoming shallower in the Southern Ocean, and thus, will likely greatly reduce suitable habitat for calcifying marine organisms with the most stressful period occurring during the austral winter months (Negrete-García *et al.*, 2019).

On the biological side, previous studies that have addressed transcriptomic responses in Antarctic ectotherms have provided similar observations of plasticity. Within Antarctic fish, exposure to the combined stress of +4 °C and low pH conditions led to a highly plastic transcriptomic response with differential expression of more than 2000 transcripts after 1 week of exposure; this response was followed by a steady decline in treatment effects, a result that potentially reflects acclimation to these conditions (Huth and Place, 2016). Other research using reciprocal transplants of benthic marine invertebrates between the subtidal and intertidal zones of the Western Antarctic Peninsula described significant plasticity in gene regulation across multiple biological pathways (Clark *et al.*, 2018); however, common garden acclimation did not lead to transcriptomic acclimation between the two populations. These studies, together with our assessment of gene expression presented here, lend support to a view that Antarctic ectotherms retain a significant degree of transcriptomic plasticity.

(d) *Description of patterns of differential gene expression*

In this section, we describe how the pteropod transcriptome changed as a function of temperature and *p*CO_2_ across the 10-day experiment. These comparisons are designed to illustrate the response and plasticity of *L. h. antarctica* to acute changes in experimental conditions. Individual pairwise assessments of DGE between pH treatments for each time point are available in supplemental file 1 and result tables are available in supplemental file 2.

*(i) Time of sampling*

**Figure 4:**
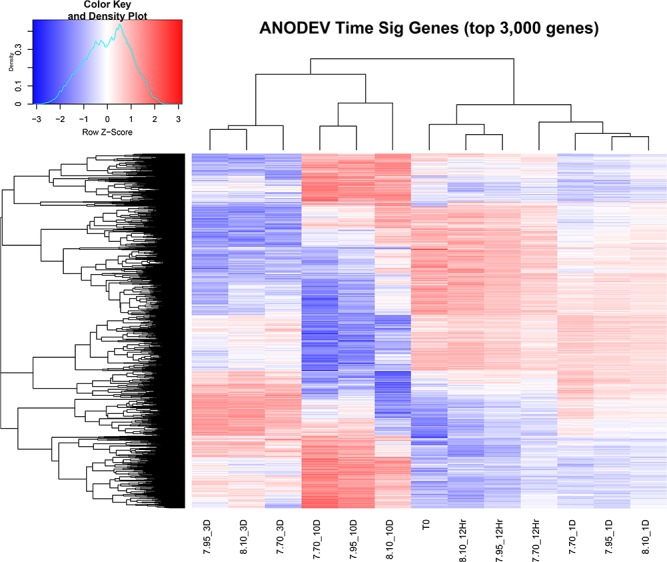
Heatmap of the top 3000 differentially expressed genes changing over time for experiment E. Sampling labeling provides treatment pH followed by sampling day, notice clustering of samples is by day first and then by treatment.

To assess the effects of sampling time for experiment E, we measured changes in gene expression between each time point (regardless of pH exposure) in reference to the pre-exposure Time 0 sample. When examining the patterns of gene expression in pteropods as a function of sampling time, we found distinct transcriptome profiles that were modified in response to the pH level in the experiment (Figure 4, Table 1). In general, there were transcriptome-wide differences within the first 12 hours that indicated there was a stress response in these animals. Specifically, transcripts associated with processes such as *oxidoreductase activity*, *nucleoside binding*, and *structural constituent of ribosome* were up-regulated while processes such as *chromatin binding*, *core promoter binding*, and *transcription corepressor* were down-regulated (see Table 1). This response transitioned between the 12 hours and Day 1 sampling and was highlighted by an enrichment of down-regulated transcripts associated with three notable processes: *identical protein binding* and *hydrolase activity* (see Table 1). By the Day 3 time-point, these transcriptome profiles were again slightly modified where enrichment of up-regulated genes associated with *peptidyl-prolyl cis-trans isomerase* and *structural constituent of ribosome* while, down-regulated transcripts were associated with *hydrolase activity* and *Ran GTPase binding* (see Table 1). Finally, on Day 10, there was an expansion among up-regulated transcripts that were enriched for gene ontologies associated with *translation regulation* and *RNA polymerase*’s *I, II,* and *III*; while the down-regulated transcripts showed enrichment for *ion binding*, *protein serine/threonine kinase*, and *hydrolase activity* (supplemental file 2)*.* Together, these broad patterns highlight an acute transcriptomic response that overtime became enriched for up-regulated RNA polymerases and translation regulators that modify gene expression pathways, while down-regulation of ion binding and *Ran GTPase binding* suggests a decline in cellular proliferation potentially associated with the observed increased rates of mortality. In addition, in light of the observed survival patterns in this experiment, we can summarize that by Day 10 we would likely be assessing the transcriptome of physiologically compromised animals.

**Figure 5:**
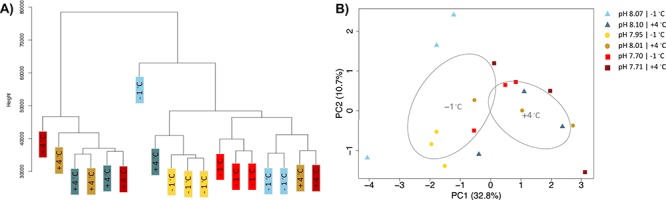
A) Hierarchical clustering of gene expression for individuals cultured at +4 °C and − 1 °C. B) Biplot of the first two principle components, sample day is represented by icon shape while pH is designated by color.

*(ii) Multiple stress data*

In order to disentangle the influence of temperature and pH on gene expression we focused on samples from one time point (24 hours) at all pH treatment in both experiments A and E (−1.2 °C and + 4 °C). Filtering of the 18 combined Day 1 libraries from both the ambient (A) and the elevated (E) temperature treatments produced a final counts matrix consisting of 49 894 transcripts with an average expression of 11.8 million reads per sample (range: 8.1–14.1 million reads). The principle coordinate analysis identified significant changes associated with temperature (Adonis p-value = 0.0006), but no effect of the interaction of temperature x pH (Adonis p-value = 0.246). Visualization of the first two principle components and the distribution of standard error on the ordination plots revealed that samples from the mid and high pH treatment in experiment A cluster together along PC1 while the samples from the low pH treatment in experiment A clustered with the samples in experiment E (Figure 5AB). These patterns highlight a transcriptomic response where gene expression in response to low pH converges on that observed for high temperature stress. Of interest is that when viewed independently of one another, these two datasets highlight the effects of pH on gene expression, but that once analyzed together the effect of low pH is not detectable over the influence of temperature – suggesting the presence of a generalized stress response in pteropods, one capable of responding to low saturation states and low pH levels, and also, the role of temperature as a “master regulator” of gene expression.

These transcriptomic profiles are interesting in that they highlight that this polar pteropod has not lost mechanisms of molecular response to variability in their abiotic environment. In addition, when speculating on the overall environmental experience of pteropods in the Ross Sea and the Southern Ocean in general (which is difficult as relatively few physical data exist for this oceanic region), it is likely that pteropods in winter do experience low pH levels now. Further, present-day aragonite undersaturation events may occur at a depth where live pteropods exist, and therefore the exposure to these conditions may not be extraordinary for modern day populations. Lastly, and very speculatively, there could be a seasonal or phenological component here as well. When considering the temperature and pH conditions pteropods encounter *in situ* we would predict that higher temperature would co-occur with high pH (in summer), fully ambient subzero temperatures with low pH (in winter), but never the combination of low pH and elevated temperature (see Kapsenberg *et al*., 2015 for time series of the physical data from McMurdo Sound). On a related note regarding this phenology, we have assessed seasonal transcriptomes in *L. h. antarctica* and found that gene expression does vary with season; here, tracking the phenology of changes in seawater conditions in McMurdo Sound and maturation of the animals (Johnson et al. 2018).

(a) *ANODEV of Ambient and acute thermal stress incubations after 24 hours*

**Figure 6:**
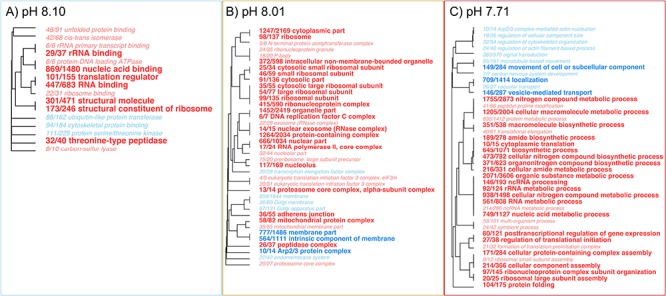
Gene ontology enrichment and term clustering of the ANODEV results comparing differential expression between the two temperature experiments (+4 °C vs. -1 °C) at each pH treatment (8.10, 8.01, 7.71). Significantly enriched gene ontologies were plotted with upregulated ontologies plotted in red text and downregulated ontologies in blue text. The boldness of the text represents FDR cut offs for significance of 0.1,0.05, and 0.01. Finally, the clustering identifies closely related ontologies.=

In order to explore how temperature influenced gene expression across pH treatments, we contrasted the ambient and elevated temperature datasets to analyze the effect of temperature at each pH level using a one-way analysis of deviance (ANODEV) in edgeR and assigned false discovery rates for multiple comparisons using the Benjamini–Hochberg procedure (Benjamini and Hochberg 1995). This approach identified 4332 transcripts that were significantly different between temperature treatments (−1 °C vs. +4 °C) in at least one of the three pH treatments (pH 8.10, 8.01 and 7.71). For each pH treatment we explored functional enrichment using the Mann-Whitney U Test with the signed-log transformed p-value for each comparison nested within the ANODEV. This approach provided a single p-value shared by all 3 comparisons forcing changes in functional enrichment to be driven by the direction of differential expression for each pH treatment.

With this approach, we observed that temperature (−1 °C vs. +4 °C) had a significant effect on gene expression with unique expression profiles for each pH treatment. Within the high pH treatment (pH 8.10) we observed significant enrichment for 96 gene ontologies including the GO terms *protein folding* and *unfolded protein binding* (Figure 6A, supplemental file 2). The mid pH treatment (pH 8.01) was enriched for transcripts associated 24 GO terms including *microtubule-based processes*, *motor activity,* and *supramolecular fibers* (Figure 6B). And the low pH treatment (pH 7.71) was composed of transcripts enriched for 106 gene ontologies with a wide range of GO terms among up-regulated transcripts associated with *ion* and *cation transmembrane transport* (Figure 4C). Of interest here is that when comparing −1 °C vs. +4 °C temperature conditions, it was only the pH treatment of pH 8.10 that showed enrichment for up-regulated transcripts associated with the GO terms *protein folding* and *unfolded protein binding*. In other words, when comparing expression in pteropods at −1 °C vs. +4 °C in the high pH (pH 8.10) treatment we observed an up-regulation of transcripts associated with maintaining proper protein structure and protein homeostasis. The fact that this response was not observed in either the mid- or low pH treatments indicates that under conditions that mimic OA, the genes involved in protein folding were no longer differentially expressed between the ambient and elevated temperature experiments. The basis for this response may reflect increased protein synthesis that occurs in summer when higher temperatures coupled with more alkaline pH conditions may cue phenological changes in gene expression associated with the seasonal patterns of pH changes, temperature, and phytoplankton abundance characteristic of the austral summer environment. In addition, this approach identified an increase in expression of ion transport and transmembrane transport transcripts observed when we compared ambient vs elevated temperatures at the low pH treatment (pH 7.71) providing reliable evidence that OW also influences intracellular ion balance. These results, in combination with the observed increased rate of mortality provides additional evidence that these two stressors (OA and OW) interact to increase the frequency of ion imbalance in what is likely a lethal manner.

**Table 2:** Differential gene expression between ambient and elevated temperatures for each pH treatment

Sequence ID	logFC pH 8.10	logFC pH 8.01	logFC pH 7.71	FDR	Gene ID
TR598442|c1_g3_i5	1.95	2.35	2.27	3.91E-05	Allene oxide synthase-lipoxygenase protein
TR614338|c0_g1_i2	3.58	3.39	−0.02	1.33E-11	Cathepsin L
TR590598|c1_g6_i1	3.09	1.50	0.03	9.86E-03	Peroxiredoxin 6
TR524084|c0_g4_i1	2.51	2.21	1.24	1.22E-06	Elongation factor 1-alpha
TR442284|c1_g3_i9	−2.97	−3.44	−3.43	2.22E-20	Von Willebrand factor type EGF and pentraxin domain-containing 1-like
TR559229|c4_g1_i24	1.40	−2.09	−0.73	0.00876473	Heat shock 70 kDa cognate 5
TR559314|c2_g2_i6	2.10	1.78	2.93	5.77E-04	Polycomb eed
TR391570|c1_g1_i3	2.40	2.00	1.75	1.80E-03	Nucleosome-remodeling factor subunit BPTF
TR580008|c0_g1_i2	2.18	1.62	1.57	1.61E-03	Dpy-30 homolog
TR382284|c3_g3_i1	−1.80	−1.81	−1.51	1.37E-02	Delta-aminolevulinic acid dehydratase-like

These effects were further explored through functional enzyme annotation of the 4332 transcripts identified in the ANODEV. This analysis identified 198 differentially expressed transcripts with Enzyme level annotation. Filtering of these transcripts revealed 9 transcripts coding for unique enzymes that were significantly differentially expressed by at least a 1.5 logFold change in all 3 pH treatments. These enzyme coding transcripts were also all differentially regulated with 6 transcripts up-regulated, and 3 transcripts down-regulated, in response to temperature. Among these were polycomb protein EED, nucleosome-remodeling factor subunit BPTF, and dpy-30, all of which were up-regulated and are predicted to influence methylation of Histone H3 ‘Lys-4’ and transcription (Table 2). There was also up-regulation of the transcript coding for allene oxide synthase-lipoxygenase, an enzyme known to be important in coral heat stress responses (Lõhelaid *et al.*, 2015). Finally, among the down-regulated enzyme-coding transcripts was the enzyme delta-aminolevulinic acid dehydratase which has a putative role in promoting protein renaturation by HSP70. The down-regulation of transcripts encoding this enzyme may reflect declining energy reserves as these organisms shift energy toward DNA replication and transcription, but away from maintaining protein structure.

Of interest, when assessing changes in transcript expression associated with molecular chaperones, we observed a global down-regulation of HSP70 transcripts. This result was further explored by plotting FPKM-adjusted counts for each library for all annotated HSP70 transcripts. This revealed that while the elevated temperature expression levels were lower at higher temperatures, they were still highly expressed FPKM >4000. In addition, these transcripts were always above an FPKM value of 3000 in all of the field samples that had been collected as part of a parallel experiment (Johnson *et al.,* 2018). One potential explanation of this is that the observed increase in HSP70 expression between the low and the high pH treatments is a reflection of increased protein synthesis under the combined conditions of high pH x elevated temperature, requiring an increase expression of molecular chaperones.

(b) *WGCNA: exploring gene expression patterns amongst the treatments*

Finally, to further describe the changes in gene expression between ambient and elevated temperature we used a WGCNA to identify gene networks associated with pH treatment and temperature. This analysis identified a total of 23 gene modules of which 4 showed significant correlation with either pH or temperature. Two of these modules were significantly correlated with temperature, one was correlated with pH treatment, and one was correlated with both temperature and pH (Fig 5 and supplemental file 3). The two modules associated with temperature were modules *turquoise* (n = 4290 transcripts) and *cyan* (n = 3247). The single largest network, module *blue* (n = 5745), was positively correlated with the interaction of temperature and pH treatment and the smallest module, module *orange* (n = 100), was positively correlated with the low pH treatment. These four modules are described in further detail below.

**Figure 7:**
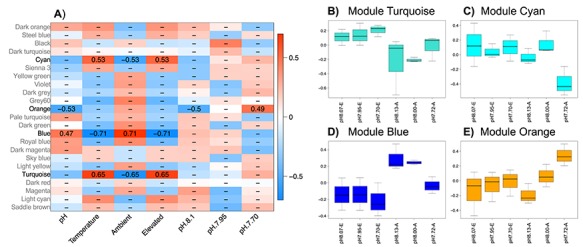
A) WGCNA network significance correlation matrix, only modules with significant correlation (P.value <0.05) are plotted and the strength and direction of correlation is provided. B-E) Displays boxplots of eigengene expression for each treatment (n = 3 samples/treatment) for each significantly expressed module. Treatments on the x-axis are identified by pH with A/E refereeing to Ambient and Elevated temperature treatments.

*(i) Modules turquoise and cyan*: Transcript expression profiles for modules *turquoise* and *cyan* were both correlated with temperature (Figure 7A) but reflect very distinct molecular functions with only a single overlapping enriched gene ontology between the two sets. Within module *turquoise* only 2015 of the 4290 transcripts have predicted functions and functional enrichment of these transcripts identified 32 enriched gene ontologies associated with *unfolded protein binding*, *oxidoreductase activity*, *cell redox homeostasis*, *the myelin sheath*, and *the voltage-gated potassium complex*. In contrast, module *cyan* contained 1251 transcripts with predicted functions and functional enrichment of 48 gene ontologies including *rRNA processing*, *rRNA* and *mRNA binding*, *the transferase complex*, *nuclear transport*, and *multiple ribosomal subunits*. The only overlapping gene ontology between these sets of transcripts was for transcripts associated with *protein folding*. We also examined KEGG enzyme enrichment in order to better understand the role each of these gene modules may be having on pteropod response to the combined stress of OA and OW. This analysis identified enrichment for 8 enzymes in module *turquoise* with an over-representation of oxidoreductases that incorporate two atoms of oxygen, peroxidases, threonine aspartase, and multiple isoforms of Allene oxide synthase-lipoxygenase’s (supplemental file 3). Module *cyan* in contrast identified enrichment for 3 enzyme terms associated with NADH dehydrogenase, NADH ubiquinone reductase, or acting on NADH/NADPH. These two modules reflect global responses to temperature stress across all pH treatments with increasing expression of transcripts associated with many ontological categories generally associated with stress response (i.e. *unfolded protein binding*, *protein folding*, and *cell redox homeostasis*). In addition, the identification of significant enrichment for allene oxide synthase-lipoxygenase’s provides further evidence that these transcripts are associated with stress response (Lõhelaid *et al.*, 2015), and may influence membrane fluidity under elevated temperatures. Identifying these two large modules that appear to respond primarily to temperature (Figure 7A-C) suggests that temperature is the major driver of transcriptomic plasticity in *L. h. antarctica*.

*(ii) Module blue*: Module *blue* was the single largest module detected by the WGCNA with 5745 transcripts being positively correlated with low temperature and high pH (Figure 7A,D). This large module was unique as it was composed of 4452 transcripts annotated with a predicted function and was enriched for 332 gene ontology terms and 24 enzymes. Enriched gene ontologies included *calcium ion binding*, *cytoskeletal protein binding*, *protein serine/threonine kinase activity*, *hydrolase activity*, and *metal ion transport*. The enriched enzymes reflect many of the enriched ontology terms with over-representation of protein-serine/threonin phosphatases, aldehyde dehydrogenase, peptidases acting on peptide bonds, histone acetyltransferases, sodium/ potassium-exchanging ATPase, and the DNA-directed DNA polymerase (supplemental file 3). The presence of so many transcripts with predicted function and enrichment for hundreds of gene ontology terms suggests this module is composed of biological processes compromised by the combined stress of OA and OW. Of interest among these groups of genes is the presences of metal ion binding and cytoskeletal binding proteins that show increased expression in the control temperature and pH conditions. This does not necessarily imply the pteropods are not actively calcifying under low pH conditions, rather these results suggest calcification may be restricted under low pH and high temperatures. One possible explanation for this is that under high pH conditions pteropods are actively calcifying, but that under low pH conditions this calcification activity is restricted to areas that are structurally compromised (see (Peck *et al.*, 2018, Peck *et al.*, 2015)), but that overall expression of genes putatively involved in calcification is reduced. Many studies seeking to further explore to what degree pteropods will be able to maintain their calcified structures under OA and OW conditions may therefore be interested in utilizing this gene network to assay stress in pteropods collected *in situ*.

*(iii) Module orange*: Finally, among all of the modules that were significantly correlated with any treatment condition, only module *orange* had a significant correlation with the pH treatments alone (Figure 7A,E). This smaller module (n = 100 transcripts) had 63 transcripts with predicted functions which were enriched for gene ontologies associated with *mRNA splicing*, *snRNA binding*, *the spliceosomal complex*, and *ribonucleoprotein complex biogenesis* (Full list supplemental file 3). This small module of genes is also of interest as the gene ontologies suggest this network maybe involved in directing transcriptional plasticity in response to low pH conditions. The presence of a molecular switch associated with declining pH may reflect the ecological history of these organisms in the Southern Ocean where annual declines in pH are associated with the austral winter. However, with the paucity of samples collected during the seasonal transition from Summer to Winter, it is currently not possible to test this hypothesis. Differential expression of transcripts associated with spliceosomal mRNA processing has also been observed in the analysis of experiment A (Johnson and Hofmann, 2017) and in the Arctic copepod, *Calanus glacialis,* following exposure to OA conditions (Bailey *et al.*, 2017). The strong correlation with pH suggests pteropods response to OA conditions may rely on alternative splicing of mRNA transcripts. Future research would benefit from the development of a reference genome for further assessing how OA and OW influence the expressed transcriptome.

## Conclusion

The data presented here provide a foundation for understanding the transcriptomic plasticity of *L. h. antarctica* to OA and OW in the future and *in situ*. Our experimental approach identified plasticity in gene expression patterns that indicated temperature was the dominant driver of DGE, and that low pH stress significantly increased transcriptomic responses. Our previous work (experiment A) has shown that in response to low pH stress alone, *L. h. antarctica* decreased gene expression for transcripts associated with biomineralization (Johnson and Hofmann 2017). This means that when confronted with environmental conditions that cause shell dissolution the organism apparently did not up-regulate genes associated with calcification, but rather, these genes become up-regulated when the organism is placed in an environment that was conducive to shell growth (i.e. high pH). This pattern was not observed under the combined stress of acidification and warming, but rather short/acute response to these multiple stressors elicited a differential heat shock response.

Because thecosome pteropods are significantly impacted by ocean acidification as a single stressor that drives shell dissolution and gene expression (Gardner *et al.,* 2017; Maas *et al.,* 2015), it is of significant interest that the Antarctic form, *L. h. antarctica*, did not increase expression of genes believed to be associated with calcification (Johnson and Hofmann, 2017). Previous assessments of gene expression changes in response to OA in other pteropods have identified increased regulation in genes associated with neuronal function and potentially calcification; however, these responses are not mimicked in the Southern Ocean form (Moya *et al.,* 2016; Johnson and Hofmann, 2017). These discrepancies may reflect the genetic divergence between the Antarctic and Arctic forms of *Limacina helicina* and other shelled pteropods (Hunt *et al.*, 2010, Sromek *et al.*, 2015), differences in experimental conditions, or differences in life history stage (Johnson *et al.,* 2018). As such, identifying the degree of plasticity in gene expression for *L. h. antarctica* to both pH and temperature stress is an important step to interpreting physiological and molecular responses.

In this study, there was minimal overlap of transcripts that were differentially expressed between the high and low pH treatments each day reflecting the sample clustering observed in the PCoA (Figure 3B). This analysis also highlights a high amount of variance between biological replicates that should have been minimized due to the fact that for each biological replicate 10 individual pteropods were pooled. This suggests that there was a wide range of variance in gene expression between individuals in the treatments, an observation that may reflect the steady increase in mortality events over time. Of interest is the breakdown of day-specific expression patterns on Day 10 at which point clustering began to separate out by pH treatment, a result that most likely drives the identification of significant influence of pH by time in the PERMANOVA analysis of variance. These findings suggest that during the first 72 hours of the experiment pteropod gene expression was primarily driven by the need to maintain cellular function under high temperatures, and that following Day 3 the acute response to thermal stress began to stabilize, after which the additive stress of acidification began to down-regulate genes associated with a broad range of gene ontologies linked to cellular replication. Additive effects of acidification and temperature have also been reported in a parallel assessment of respiration rates to the multi-stressor experiment presented by Hoshijima *et al.* (2017). This decline in transcript expression across so many ontological terms may reflect the increased rate of mortality under the combined stress and as such we will focus the remainder of this discussion on changes in expression after 12, 24 and 72 hours of exposure to the dual stressors.

Overall, the acute response to the combined stressors was the most pronounced in the low pH treatment where heat shock protein transcripts and general molecular chaperone transcripts were up-regulated following 12 hours of exposure. These up-regulated HSP70 cognate 5 (logFC = +4.20) and dnaJ homolog (logFC = +6.8) transcripts suggest that the combined stressors did activate the cellular stress response. Differential regulation of transcripts within these gene categories are evidence that there is additional stress on maintaining protein folding from acidified conditions that is not present in the high pH treatment, leading to up-regulation of these HSP70 transcripts that were also constitutively expressed in all treatments. Previous assessments of thermal tolerance in Antarctic invertebrates have highlighted that HSP70 transcripts do not exhibit differential expression in response to thermal stress (González *et al.*, 2016, González-Aravena *et al.*, 2018). In contrast, Huth and Place (2016) found a modest increase in expression among a few HSP transcripts when notothenioid fish were exposed to OA conditions that were similar to those used in this study. Together these transcriptome-wide assessments of gene expression in response to OA and OW support previous observations that the majority of HSP transcripts exhibit constitutive expression.

Numerous studies on Antarctic marine invertebrates have explored the adaptive capacity to ocean warming (Peck, Morley, Richard and Clark, 2014) and identified a decline in thermal tolerance with age (Peck, Souster and Clark, 2013). Two competing explanations for this limited response to thermal stress suggest that the elevated constitutive expression of these molecular chaperones is either: i) important for repairing cold-induced protein denaturation (Place *et al.*, 2004, Todgham *et al.*, 2007) or, ii) that these chaperones are simply less effective at low temperatures requiring a constitutively high level of production to maintain proper protein folding at all times Logan and Buckley (2015). While it is still not possible to distinguish which hypothesis is valid, our data provide evidence that pteropods are able to up-regulate a small proportion of molecular chaperones when exposed to the combined stressors of OA and OW suggesting that the constitutively expressed chaperones may already be involved in repairing protein denaturation under ambient conditions. In addition, our analysis identified an up-regulation of Allene oxide synthase-lipoxygenase’s between experiment’s A and E and over time within experiment E when compared to the time 0. These results along with studies in coral systems (Lõhelaid *et al.,* 2015) provide evidence that the Allene oxide synthase-lipoxygenase’s may function in the cellular stress response and influence membrane composition.

From a broader perspective, polar pteropods are of interest as they will be among the first impacted by climate change in high latitude seas (Henley *et al.*, 2019, Manno *et al.*, 2017). Along the West Antarctic Peninsula, declining sea ice cover and ocean warming have strong influences on macrozooplankton abundances and distributions (Henley *et al.*, 2019, Steinberg *et al.*, 2015). Such effects have been observed in McMurdo Sound where variation in seasonal food quality has been associated with differential responses to OA and gene expression in *L. h. antarctica* (Johnson *et al.*, 2018; Seibel *et al.*, 2012). In context of the experiment presented here, it is plausible that the variation in mortality between the ambient and elevated temperature experiments may be reflective of difference in energy reserves between the two collections.

Finally, these experiments have further highlighted the vulnerability of *L. h. antarctica* to these multi-stressors and provided transcriptomic evidence that temperature is the master variable driving global changes in gene expression. And further, that although *L. h. antarctica* did display some plasticity here, future studies on how the plasticity of genomic biology and gene expression will continue to add perspective to adaptation of ectotherms of life in Antarctic waters, and whether theses polar organisms have the capacity to respond and tolerate future oceanic pH and temperatures.

## Funding

This work was supported by a grant from the U.S. National Science Foundation (NSF) to GEH (PLR-1246202). In addition, KMJ was supported by a U.S. NSF Graduate Research Fellowship under grant no. 1650114.

## Supplementary Material

Supplemental_File_1_coaa013

Supplemental_File_2_coaa013

Supplemental_File_3_coaa013

Supplemental_File_4_coaa013
