# Supplementary material for: Combined stress of ocean acidification and warming influence survival and drives differential gene expression patterns in the Antarctic pteropod, Limacina helicina antarctica
Source: Conserv Physiol. 2020 Mar 26;8(1):coaa013. doi: 10.1093/conphys/coaa013 (PMC7098371; doi:10.1093/conphys/coaa013)
Supplement: Supplemental_File_1_coaa013 [file supplemental_file_1_coaa013.docx]

***Supplemental Results***

1. *Mortality Results Experiment E* (+4 °C)

| Sample | pH | Day | Dead | Alive |
| --- | --- | --- | --- | --- |
| 8.1_0 | 8.1 | 0 | 0 | 800 |
| 8.1_0 | 8.1 | 0 | 0 | 800 |
| 8.1_0 | 8.1 | 0 | 0 | 800 |
| 7.95_0 | 7.95 | 0 | 0 | 800 |
| 7.95_0 | 7.95 | 0 | 0 | 800 |
| 7.95_0 | 7.95 | 0 | 0 | 800 |
| 7.71_0 | 7.71 | 0 | 0 | 800 |
| 7.71_0 | 7.71 | 0 | 0 | 800 |
| 7.71_0 | 7.71 | 0 | 0 | 800 |
| 8.1_1 | 8.1 | 1 | 0 | 600 |
| 8.1_1 | 8.1 | 1 | 0 | 600 |
| 8.1_1 | 8.1 | 1 | 0 | 600 |
| 7.95_1 | 7.95 | 1 | 0 | 600 |
| 7.95_1 | 7.95 | 1 | 0 | 600 |
| 7.95_1 | 7.95 | 1 | 0 | 600 |
| 7.71_1 | 7.71 | 1 | 0 | 600 |
| 7.71_1 | 7.71 | 1 | 0 | 600 |
| 7.71_1 | 7.71 | 1 | 0 | 600 |
| 8.1_2 | 8.1 | 2 | 0 | 600 |
| 8.1_2 | 8.1 | 2 | 0 | 600 |
| 8.1_2 | 8.1 | 2 | 0 | 600 |
| 7.95_2 | 7.95 | 2 | 0 | 600 |
| 7.95_2 | 7.95 | 2 | 0 | 600 |
| 7.95_2 | 7.95 | 2 | 0 | 600 |
| 7.71_2 | 7.71 | 2 | 0 | 600 |
| 7.71_2 | 7.71 | 2 | 0 | 600 |
| 7.71_2 | 7.71 | 2 | 0 | 600 |
| 8.1_3 | 8.1 | 3 | 0 | 500 |
| 8.1_3 | 8.1 | 3 | 0 | 500 |
| 8.1_3 | 8.1 | 3 | 0 | 500 |
| 7.95_3 | 7.95 | 3 | 0 | 500 |
| 7.95_3 | 7.95 | 3 | 0 | 500 |
| 7.95_3 | 7.95 | 3 | 0 | 500 |
| 7.71_3 | 7.71 | 3 | 0 | 500 |
| 7.71_3 | 7.71 | 3 | 0 | 500 |
| 7.71_3 | 7.71 | 3 | 0 | 500 |
| 8.1_4 | 8.1 | 4 | 0 | 500 |
| 8.1_4 | 8.1 | 4 | 0 | 500 |
| 8.1_4 | 8.1 | 4 | 0 | 500 |
| 7.95_4 | 7.95 | 4 | 0 | 500 |
| 7.95_4 | 7.95 | 4 | 0 | 500 |
| 7.95_4 | 7.95 | 4 | 0 | 500 |
| 7.71_4 | 7.71 | 4 | 0 | 500 |
| 7.71_4 | 7.71 | 4 | 0 | 500 |
| 7.71_4 | 7.71 | 4 | 0 | 500 |
| 8.1_5 | 8.1 | 5 | 0 | 400 |
| 8.1_5 | 8.1 | 5 | 0 | 400 |
| 8.1_5 | 8.1 | 5 | 0 | 400 |
| 7.95_5 | 7.95 | 5 | 0 | 400 |
| 7.95_5 | 7.95 | 5 | 0 | 400 |
| 7.95_5 | 7.95 | 5 | 0 | 400 |
| 7.71_5 | 7.71 | 5 | 0 | 400 |
| 7.71_5 | 7.71 | 5 | 0 | 400 |
| 7.71_5 | 7.71 | 5 | 0 | 400 |
| 8.1_6 | 8.1 | 6 | 0 | 400 |
| 8.1_6 | 8.1 | 6 | 0 | 400 |
| 8.1_6 | 8.1 | 6 | 0 | 400 |
| 7.95_6 | 7.95 | 6 | 0 | 400 |
| 7.95_6 | 7.95 | 6 | 0 | 400 |
| 7.95_6 | 7.95 | 6 | 0 | 400 |
| 7.71_6 | 7.71 | 6 | 0 | 400 |
| 7.71_6 | 7.71 | 6 | 0 | 400 |
| 7.71_6 | 7.71 | 6 | 0 | 400 |
| 8.1_7 | 8.1 | 7 | 0 | 400 |
| 8.1_7 | 8.1 | 7 | 0 | 400 |
| 8.1_7 | 8.1 | 7 | 0 | 400 |
| 7.95_7 | 7.95 | 7 | 0 | 400 |
| 7.95_7 | 7.95 | 7 | 0 | 400 |
| 7.95_7 | 7.95 | 7 | 0 | 400 |
| 7.71_7 | 7.71 | 7 | 0 | 400 |
| 7.71_7 | 7.71 | 7 | 0 | 400 |
| 7.71_7 | 7.71 | 7 | 0 | 400 |
| 8.1_8 | 8.1 | 8 | 0 | 400 |
| 8.1_8 | 8.1 | 8 | 0 | 400 |
| 8.1_8 | 8.1 | 8 | 0 | 400 |
| 7.95_8 | 7.95 | 8 | 0 | 400 |
| 7.95_8 | 7.95 | 8 | 0 | 400 |
| 7.95_8 | 7.95 | 8 | 0 | 400 |
| 7.71_8 | 7.71 | 8 | 0 | 400 |
| 7.71_8 | 7.71 | 8 | 0 | 400 |
| 7.71_8 | 7.71 | 8 | 0 | 400 |
| 8.1_9 | 8.1 | 9 | 7 | 393 |
| 8.1_9 | 8.1 | 9 | 5 | 388 |
| 8.1_9 | 8.1 | 9 | 4 | 384 |
| 7.95_9 | 7.95 | 9 | 21 | 363 |
| 7.95_9 | 7.95 | 9 | 10 | 353 |
| 7.95_9 | 7.95 | 9 | 12 | 341 |
| 7.71_9 | 7.71 | 9 | 23 | 318 |
| 7.71_9 | 7.71 | 9 | 21 | 297 |
| 7.71_9 | 7.71 | 9 | 20 | 277 |
| 8.1_10 | 8.1 | 10 | 3 | 297 |
| 8.1_10 | 8.1 | 10 | 2 | 295 |
| 8.1_10 | 8.1 | 10 | 4 | 291 |
| 7.95_10 | 7.95 | 10 | 8 | 283 |
| 7.95_10 | 7.95 | 10 | 3 | 280 |
| 7.95_10 | 7.95 | 10 | 6 | 274 |
| 7.71_10 | 7.71 | 10 | 5 | 269 |
| 7.71_10 | 7.71 | 10 | 6 | 263 |
| 7.71_10 | 7.71 | 10 | 5 | 258 |

1. **PCA of all experiment E and day 1 of experiment A, also includes both Time Zero samples.**

(iii) *Experiment E:* *Pairwise DGE after 12 Hours*: Pairwise assessments of differential gene expression during the first 12 hours of acute exposure to +4 °C identified a total of 3,324 transcripts that were differentially regulated in any of the 3 pair-wise comparisons between the pH treatments (Table/Fig 2). Functional enrichment of the differentially expressed transcripts in low pH when compared to high pH treatment revealed an up-regulation of transcripts (n=893) associated with *chaperone binding*, *HSP90 binding*, and *general protein folding*; while, down-regulated transcripts (n=1,448) were associated with *calcium ion binding*, *motor activity*, *fatty acid beta-oxidation*, and *protein peptidyl-prolyl isomerization*. In contrast, when comparing up-regulated transcripts between the low and mid pH treatments (n=603) there was enrichment for transcripts associated with *protein folding*; but, no significant changes in expression among the *chaperone binding* or *HSP90 binding* gene ontologies. However, enrichment analysis among the down-regulated transcripts in the same low-mid pH comparison (n=784) identified 8 enriched gene ontologies, all of which were members of large broad gene ontologies containing greater than 1,800 transcripts in the reference set, these included *nucleoside-triphosphatase activity*, *cellular process*, and *intracellular organelle group* terms. These patterns are reflective of what was observed in the acute pH response observed under ambient conditions (Johnson and Hofmann 2018). Of interest here is the increased expression levels for chaperone binding and protein folding gene ontologies in the low pH treatments, and the lack of this response between the low and mid pH treatments. This supports previous findings showing that the combined stress of temperature and pH have an additive effect on respiration rate (Hoshijima et al. 2018). Up-regulation of genes associated with protein folding in both comparisons (low vs mid and low vs high pH) suggest that maintaining proper protein structure is especially challenged under low pH conditions when combined with elevated temperature stress.

(iv) *Experiment E*: *Pairwise DGE after 24 Hours*: Assessment of differential gene expression following 24 hours of exposure identified 2,594 differentially expressed transcripts between all treatments. Functional enrichment of the differentially expressed transcripts between the low pH and high pH treatments revealed increased expression, in low pH conditions, of 679 transcripts associated with *protein de-ubiquitination*, *epigenetic regulation of gene expression*, and *maintenance of protein location*. Down-regulated transcripts (n=673) following the 24 hours of exposure had functional enrichment for only broad gene ontology terms (> 4,000 transcripts in ontological term). Enrichment analysis among up-regulated genes in the low pH treatment when compared to the mid pH treatments (n=659) identified 16 GO terms with 5 terms in narrow gene ontologies associated with *protein folding*, *protein localization*, *unfolded protein binding*, and *small GTPase mediated signal transduction*. Once again, the 709 down-regulated transcripts between the mid pH and low pH treatments were enriched for 9 broad gene ontology terms associated with *intracellular membrane-bound organelles*, *metabolic processes*, and *catalytic activity*. This 24-hour time-point reflects major shifts in gene expression as pteropods in every treatment dramatically modify their transcriptomes.

To further explore this pattern, we assessed transcript expression between the 12-hour and 24-hour time-points across all three treatments using an ANODEV that identified 13,874 significantly variable transcripts. This suggests that the variation in gene expression between treatments identified divergent pathways that pteropods in each pH condition are utilizing in an attempt to acclimate to these dramatic changes in pH and temperature. The enrichment for *maintaining protein location* and *protein deubiquitination* suggest that the unfolded protein response observed in the first 24 hours has shifted to sequestering, editing and/or disassembly of degraded protein intermediates in the low pH condition. However, when comparing the low and mid pH treatments we observe a similar up-regulation among genes associated with *protein folding* and *unfolded protein binding* similar to what was observed between the low and high pH treatments during the acute 12-hour exposure. One additional source of variation between these two time-points that is potentially influencing the magnitude of differential expression in this case is circadian rhythm that may control daily changes in gene expression. All additional sampling days were conducted at the same time of each day to minimize this influence.

(v) *Experiment E*: *Pairwise DGE after 72 Hours*: Pair-wise differential gene expression analysis following 72 hours identified 3,365 differentially expressed transcripts between all treatments. Functional enrichment of the differentially expressed transcripts between the low pH versus the high pH treatments revealed enrichment of up-regulated transcripts (n=1,061) associated with *mRNA processing*, *regulation of gene expression*, *RNA splicing*, *rRNA processing*, and *structural components of ribosomes*. Functional enrichment of the down-regulated transcripts (n=1,057) from this low vs. high pH comparison identified 19 enriched gene ontologies associated with *protein folding*, *helicase activity*, *oxidoreductase activity*, and *unfolded protein binding*. Enrichment of gene ontologies among up-regulated transcripts between the low and mid pH treatments (n=1,082) identified 40 enriched ontologies that included, *unfolded protein binding*, *protein stabilization*, *histone modification*, and *response to stress*. Down-regulated transcripts (n=858) within this comparison identified 14 enriched gene ontological terms associated with *chromosome organization*, *elongator holoenzyme complex*, *U1 snRNPs, U2-type pre-splicesosome,* and *DNA binding*. This 72-hour time-point was taken in order to assess gene expression changes associated with pH and temperature stress after pteropods were predicted to have started to acclimate to the acute stress. Comparing the low pH to the high pH treatments identified increased expression among genes associated with increasing gene expression, while comparisons with the mid pH treatment identify increased expression of genes associated with stress responses. These differences suggest pteropods in the low pH treatment are more challenged by the combination of pH and temperature than individuals in either the mid or high pH treatments. Of interest here is the differences in expression between the mid and the high pH treatment. Specifically, we observed increased expression in the high pH treatment of genes associated with *cell proliferation*, *anatomical structure* *morphogenesis*, and the *endomembrane system*. The increase in gene expression within the high-pH treatment suggests that pteropods have acclimated to the acute exposure to both stressors and have re-invested in cellular proliferation and growth.

(vi) *Experiment E*: *Pairwise DGE after 240 Hours*: The final assessment of differential gene expression following 240 hours identified 9,859 differentially expressed transcripts between all treatments. Functional enrichment of the differentially expressed transcripts between the low pH and high pH treatments revealed 2,386 transcripts were up-regulated but were associated with only a single gene ontology, *protein folding*. In contrast, the down-regulated genes (n=6,552) were enriched for 223 gene ontologies, the ontologies with the highest numbers of genes present in the down-regulated gene set included, *calcium ion binding*, *protein serine/threonine kinase activity*, *extracellular exosome*, and *ubiquitin-dependent protein catabolic processes*. Enrichment of gene ontologies among up-regulated transcripts between the low pH and mid pH treatments (n=786) identified enrichment of 11 ontologies associated with *RNA processing*, *coenzyme binding*, *transferase complex*, and *citrate metabolic processes*. Enriched gene ontologies among the down-regulated transcripts (n=1,322) included, *mRNA processing*, *transcription regulator activity*, *magnesium ion binding*, and *regulation of Ras-protein signal transduction*. This final time-point further highlights the inability for pteropods in the low pH treatment to acclimate to the combined stress of pH and temperature. The continual elevated expression of genes associated with *protein folding* at the low pH condition and the dramatic down-regulation of 223 gene ontologies potentially reflects the increased mortality among the low pH treatment. While these data are impacted by the lack of active feeding within the treatment containers, the enrichment for only a single gene ontology among the up-regulated transcripts provides further support that at elevated temperatures low pH stress is further driving up the expression of transcripts associated with maintaining proper protein structure.
